# Increased Treg in Kidney Transplant Recipients With Erythrocytosis

**DOI:** 10.3389/ti.2025.15845

**Published:** 2025-11-25

**Authors:** Carolina Bigatti, Sadia Mustofa, Dana Korogodsky, Yorg Azzi, Elie Salloum, Andrea Angeletti, Enver Akalin, Maria Ajaimy, Paolo Cravedi

**Affiliations:** 1 Translational Transplant Research Center (TTRC), Department of Medicine, Renal Division, Icahn School of Medicine at Mount Sinai, New York, NY, United States; 2 Nephrology, Dialysis and Transplantation Unit, IRCCS Istituto Giannina Gaslini, Genoa, Italy; 3 Montefiore Medical Center, Albert Einstein College of Medicine Abdominal Transplant Center, Bronx, NY, United States; 4 University of Heidelberg, Heidelberg, Germany

**Keywords:** PTE, regulatory T cells, monocytes, EPO, erythropoietin

Dear Editors,

Erythropoietin (EPO) is a glycoprotein hormone produced predominantly by the kidney in response to hypoxia. While widely known for its hematopoietic role, EPO also exerts immune-modulating effects. In murine transplant models, administration of exogenous EPO prolongs allograft survival by increasing the frequency of regulatory T cell (Treg) and by promoting macrophage polarization towards an anti-inflammatory phenotype [1]. Consistent with these preclinical findings, clinical studies have demonstrated that recombinant EPO administration at the doses normally used to correct anemia in humans enhances both the number and suppressive capabilities of circulating Tregs [2, 3].

The immune-modulating effects of endogenous EPO in kidney transplant recipients are less clearly defined. Mice that are EPO deficient are prone to the development of autoimmunity. Patients with chronic kidney disease (CKD) often show intrarenal immune infiltrates, which may be due, at least in part, to the reduced EPO production associated with impaired kidney function. However, CKD patients exhibit multiple inflammatory sources, complicating any direct causal relationship between reduced endogenous EPO levels and intrarenal inflammation.

To more rigorously investigate endogenous EPO’s immunological impact in humans, we focused on kidney transplant recipients with post-transplant erythrocytosis (PTE), a complication affecting approximately 10%–20% of patients, typically within the first 2 years post-transplant. PTE is characterized by persistently elevated hematocrit levels without ongoing blood loss, hypoxia, or exogenous EPO therapy [4]. We hypothesized that kidney transplant recipients (KTRs) with PTE have high EPO levels and exhibit a distinct immunological profile, characterized by increased circulating Tregs and monocytes with an anti-inflammatory profile.

We conducted a cross-sectional study of 14 KTRs with PTE (hematocrit ≥50%) and 19 matched controls without PTE. Using flow cytometry, we quantified circulating immune subsets, including regulatory T cells (Tregs), T cells, B cells, and monocytes. Cytokine levels were assessed by ELISA (see Supplementary Material).

Patients with PTE and controls were similar in terms of age (52.9 ± 11.5 vs. 52.1 ± 10.5 years, *p* = 0.8), time since transplantation (4.1 ± 2.6 vs. 5.6 ± 1.9 years, *p* = 0.08), and kidney function (creatinine 1.4 ± 0.4 vs. 1.4 ± 0.5 mg/dL, *p* = 0.8). Consistent with prior reports [5], the PTE group had a significantly higher proportion of male patients (92.9% vs. 52.6%, *p* = 0.02). No significant differences were observed in induction therapy, donor-specific antibodies (DSA), or prior rejection episodes. The distribution of race/ethnicity and underlying kidney disease was comparable between groups (Supplementary Table S1). The use of RAS inhibitors (Renin-Angiotensin system inhibitors) was comparable between PTE patients and controls. Secondary causes of erythrocytosis, including renal artery stenosis and renal tumors, were ruled out in PTE patients. Additional cancer screening was not performed when PTE diagnosis occurred shortly after transplantation, considering the limited time for malignancy development. Patients with PTE had significantly higher serum EPO levels compared to controls (10.5 ± 5.9 vs. 6.8 ± 2.5 mIU/mL, *p* = 0.02) (Supplementary Figure S1A).

Tregs, defined phenotypically as CD4^+^CD25^+^CD127^low^ cells, were significantly higher in patients with PTE compared to controls (3.4% ± 1.3% vs. 2.1% ± 1.3, respectively; *p* = 0.0096) (Figures 1A,B). We did not observe significant differences in the overall percentages of CD4^+^ and CD8^+^ T cells, or B cells, between PTE patients and controls (Figure 1C). Intracellular cytokine analysis of CD4^+^, CD8^+^ T cells (including IL-17, IL-4, IFN-γ) and B cells did not reveal significant differences between the two groups.

**FIGURE 1:**
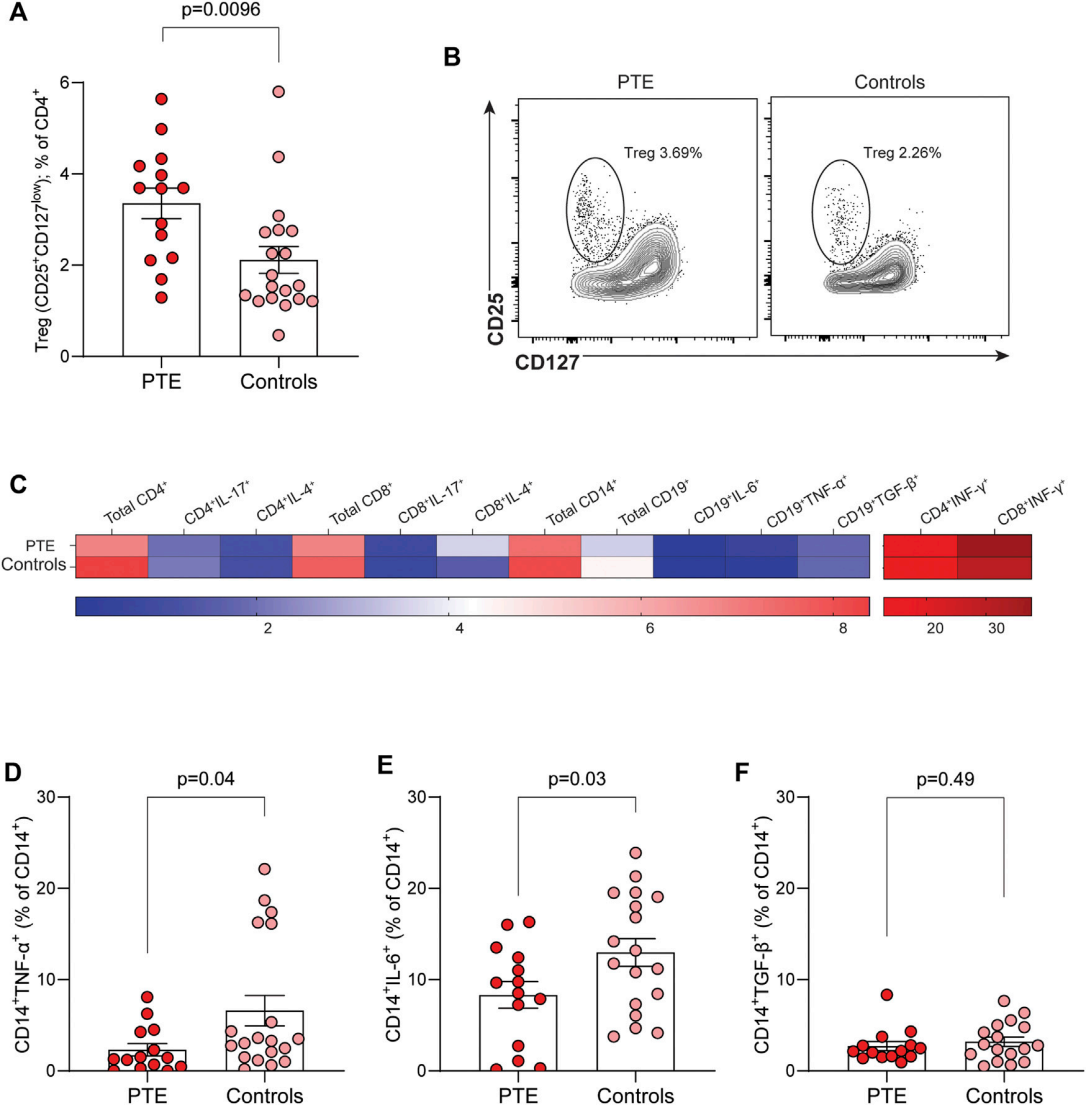
Immune profile in PTE and control kidney transplant recipients. **(A)** Frequency of Treg (CD4^+^CD25^+^CD127^low^) among CD4^+^ T cells in the two study groups. **(B)** Representative flow cytometry plots. **(C)** Heatmap showing mean frequencies of total and cytokine-expressing CD4^+^, CD8^+^ T cells, monocytes (CD14^+^), and B cells (CD19^+^) in PTE patients (n = 14) and controls (n = 19), expressed as percentage of parent population. Frequencies of TNF-α^+^ **(D)**, IL-6^+^ **(E)**, and TGF-β^+^ **(F)** monocytes (CD14^+^) following *ex vivo* LPS stimulation. Data are shown as mean ± SEM. Statistical comparisons were performed using unpaired, two-tailed t-tests.

Monocyte percentages did not differ between PTE patients and controls (Figure 1C), but functional analyses revealed that, upon stimulation with LPS (5 ng/mL), the percentages of monocytes producing TNF-α (2.3% ± 2.5% vs. 6.6% ± 7.3, respectively; *p* = 0.043) and IL-6 (8.3% ± 5.5% vs. 12.9% ± 6.4, respectively; *p* = 0.038) were significantly lower in PTE patients compared to controls. TGF-β production in monocytes did not differ between the two study groups (Figures 1D–F).

Plasma levels of TNF-α (22.11 vs. 22.56 pg/mL; p = 0.92) and IL-6 (10.79 vs. 12.65 pg/mL; p = 0.69) were comparable between PTE patients and controls (Supplementary Figures S1B, C).

In summary, we found that elevated EPO levels in KTRs with PTE are associated with increased frequency of circulating Treg and a reduced proinflammatory activation profile in monocytes, despite comparable systemic levels of TNF-α and IL-6. These findings suggest that elevated endogenous EPO may promote a more tolerogenic immune environment after kidney transplantation.

Published data by our group and others indicate that EPO promotes the release of active TGF-β by monocytes/macrophages, promoting conversion of naive CD4^+^ T cells into functional Treg [1, 6]. We also showed that EPO induces an anti-inflammatory program in macrophages, although the molecular mechanisms are not fully clear.

The combination of increased Tregs and reduced monocyte-derived inflammation in PTE patients raises the intriguing possibility that this cohort of patients may be at lower risk of acute rejection due to the immune-regulatory effects of EPO [7]. On the other hand, the recent evidence that tumors producing EPO have lower chances to be cleared by the anti-tumor immune response [8], supports studies testing the neoplastic risk of these patients.

We also observed a lower rate of DSA in PTE patients (7% vs. 31% in controls). Although this difference did not reach statistical significance, this trend may be biologically relevant, suggesting a possible protective role of EPO against alloimmune sensitization, as we previously reported in mice [9].

Our study has several limitations, including its relatively small sample size and cross-sectional design, which limit the ability to infer causality. Additional prospective studies with larger cohorts are required to confirm our findings, elucidate the precise mechanisms by which endogenous EPO modulates immune responses in humans, and determine the long-term implications for graft outcomes.

In conclusion, consistent with animal studies and previous clinical data involving recombinant EPO, our findings suggest that elevated endogenous EPO levels in kidney transplant recipients with PTE promote an anti-inflammatory immune environment under stable immunosuppression. This evidence could be leveraged to test the hypothesis that, in this patient population, lower levels of immunosuppression are needed to prevent rejection. On this line, an ongoing prospective study is testing the hypothesis that EPO administration allows safe immunosuppression withdrawal in stable liver transplant recipients (NCT06832189).

## Data Availability

The raw data supporting the conclusions of this article will be made available by the authors, without undue reservation.
