# Supplementary material for: Increased Treg in Kidney Transplant Recipients With Erythrocytosis
Source: Transpl Int. 2025 Nov 25;38:15845. doi: 10.3389/ti.2025.15845 (PMC12685754; doi:10.3389/ti.2025.15845)
Supplement: Supplementary file 1 [file DataSheet1.pdf]

Supplementary Methods

Study design

This was a cross-sectional observational study conducted at the Montefiore Transplant Center, New York, US, between April 2024 and February 2025. We enrolled adult kidney transplant recipients with post-transplant erythrocytosis (PTE), defined as hematocrit ≥50% in the absence of exogenous EPO therapy or ongoing bleeding. Patients without PTE (hematocrit <50%) served as controls.

From April 2024 to February 2025, we enrolled 14 KTRs with PTE (hematocrit ≥50%) and 19 without PTE (hematocrit <50%) at the Montefiore Transplant Center, New York, US. Control patients were matched to PTE cases based on transplant date (±4 weeks).

The primary endpoint was the percentage of circulating Treg in PTE and non-PTE recipients. Secondary endpoints included the EPO level, percentages of the other major circulating immune cell populations, and plasma cytokine concentrations in PTE and non-PTE recipients.

PBMC staining and stimulation

PBMC were isolated from peripheral blood by Ficoll gradient and frozen for batched analysis. Treg cells were identified on unstimulated PBMCs using surface markers ($CD4^{+}CD25^{+}CD127^{low)}$. For cytokine analysis, PBMCs were stimulated for 3 hours at 37 °C in complete RPMI medium in the presence of Golgi Plug (BD Biosciences) to allow intracellular cytokine accumulation.

T cells were activated with phorbol 12-myristate 13-acetate (PMA, 100 ng/mL) and ionomycin (2 μg/mL).

B cells and monocytes were stimulated with lipopolysaccharide (LPS, 5 ng/mL) and ionomycin (2 µg/mL).

Following stimulation, cells were stained for extracellular markers and intracellular cytokines (IFN-γ, IL-4, IL-17 for T cells and TNF-α, IL-6, TGF-β for B cells and monocytes).

Flow Cytometry

We designed three multicolor flow cytometry panels to quantify Treg, T cells, B cells and monocytes and the intracellular cytokine production. The following fluorochrome conjugated anti-human antibodies were used from BD Biosciences (San Jose, CA): CD3-PerCP-Cy5.5, CD8-BV650, CD19-BV510, IFN-γ-PE-Cy7, IL-4-PE, TNF-α-PE, IL-6-APC, TGF-β-BV421; from Biolegend (San Diego, CA): CD4-APC-Cy7, CD127-FITC, CD27-APC; from Invitrogen (Waltham, MA): CD4-PE-Cy7, IL-17A-APC; from Beckman Coulter (Brea, CA):CD14-FITC. Data were acquired on a three-laser Canto II flow cytometer (BD Biosciences) and analyzed using FlowJo (https:// www.flowjo.com) software.

ELISA

Plasma erythropoietin concentrations were measured using the Human Erythropoietin SimpleStep ELISA Kit (Abcam, ab274397), following the manufacturer’s instructions.

Plasma levels of TNF-α and IL-6 were quantified using precoated Human ELISA kits from Invitrogen (TNF-αKAC1751 and IL-6: KAC1261, respectively).

All samples were assayed in duplicate, and absorbance was measured at 450 nm using a microplate reader. Cytokine concentrations were calculated using standard curves generated from recombinant protein standards.

Statistical Analysis

Continuous data were stated as mean ± standard deviation or medians and interquartile ranges. Variables were compared by unpaired t-test. Categorical variables were stated as count and percentage and compared between the two conditions using a chi-squared test or Fisher's exact test, as appropriate. All P-values were two-sided, and $P<0.05$ was considered statistically significant. Data were analyzed using GraphPad Prism 10.

Table S1. Baseline patients' characteristic.

	PTE (n=14)	Controls (n=19)	p value
**Age,** years	52.9 ± 11.5	52.1 ±10.5	0.8
**Male gender**, n (%)	13 (92.9)	10 (52.6)	0.02
**Hematocrit,** %	52.3 ± 2.3	38.9 ±6.5	<0.0001
**Time after Transplant**, years	4.1 ± 2.6	5.6 ± 1.9	0.08
**Race/ethnicity**, n (%)			0.72
African American	5 (35.7)	7 (36.8)	
Hispanic	8 (57.1)	9 (47.4)	
White	1 (7.1)	3 (22.2)	
**Kidney disease**, n (%)			0.97
Diabetic nephropathy	6 (42.9)	7 (33.3)	
Hypertensive nephropathy	3 (21.4)	5 (22.2)	
Glomerulonephritis	4 (28.6)	6 (33.3)	
Others	1 (7.1)	1 (5.6)	
**Induction**, n (%)			0.7
Thymoglobulin	10 (71.4)	15 (79)	
Basiliximab	4 (28.6)	4 (21)	
**DSA**, n (%)	1 (7.1)	6 (31.6)	0.2
**Prior rejection**, n (%)	1 (7.1)	2 (10.53)	>0.999
**Creatinine**, mg/dl	1.4 ± 0.4	1.4 ± 0.5	0.8
**ACEi/ARBs**, n (%)	8 (57.4)	6 (28.5)	0.09
**Phlebotomy**, n (%)	1 (7.1)	1 (5.26)	0.8

DSA: Donor-Specific Antibodies; ACEi: Angiotensin-Converting Enzymes inhibitors; ARBs: Angiotensin II Receptor Blockers.

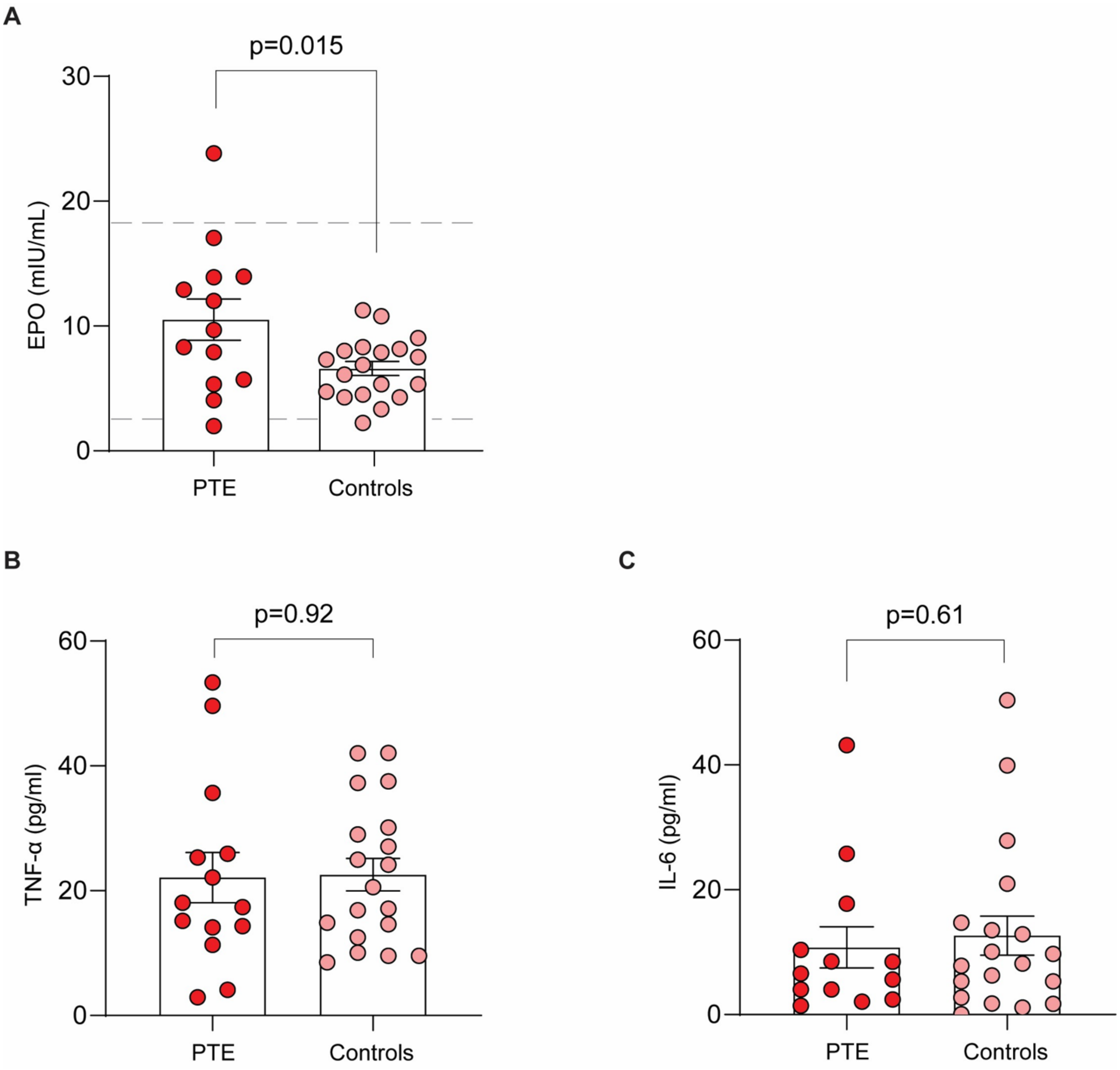

Figure S1. (A) Plasma EPO concentrations in PTE patients (n = 14) and controls (n = 19), measured by ELISA. Dotted line indicates the lower and the upper limit of the normal range. Plasma concentrations of TNF-α (B) and IL-6 (C), measured by ELISA. Data are presented as mean ± SEM.
